# Age-Related Differences in Cortical Activity during a Visuo-Spatial Working Memory Task with Facial Stimuli

**DOI:** 10.1371/journal.pone.0075778

**Published:** 2013-09-19

**Authors:** Flávia Schechtman Belham, Corina Satler, Ana Garcia, Carlos Tomaz, Antonella Gasbarri, Artur Rego, Maria Clotilde H. Tavares

**Affiliations:** 1 Laboratory of Neuroscience and Behavior, Department of Physiological Sciences, University of Brasilia, Brasilia, Brazil; 2 Faculty of Ceilandia, University of Brasilia, Brasilia, Brazil; 3 Department of Biomedical Sciences and Technologies, University of L'Aquila, L'Aquila, Italy; Roma Tre University, Italy

## Abstract

Emotion, importantly displayed by facial expressions, is one of the most significant memory modulators. The interaction between memory and the different emotional valences change across lifespan, while young adults (YA) are expected to better recall negative events (Negativity Bias Hypothesis), older adults (OA) tend to focus on positive stimuli (Positivity Effect Hypothesis). This research work aims at verifying whether cortical electrical activity of these two age groups would also be differently influenced by emotional valences in a visuo-spatial working memory task. 27 YA (13 males) and 25 OA (14 males), all healthy volunteers, underwent electroencephalographic recordings (21 scalp electrodes montage), while performing the Spatial Delayed Recognition Span Task using a touch screen with different stimuli categories: neutral, positive and negative faces and geometric pictures. YA obtained higher scores than OA, and showed higher activation of theta and alpha bands in the frontal and midline regions, besides a more evident right-hemispheric asymmetry on alpha band when compared to OA. For both age groups, performance in the task was worse for positive faces than to negative and to neutral faces. Facial stimuli induced a better performance and higher alpha activation on the pre-frontal region for YA, and on the midline, occipital and left temporal regions for OA when compared to geometric figures. The superior performance of YA was expected due to the natural cognitive deficits connected to ageing, as was a better performance with facial stimuli due to the evolutionary importance of faces. These results were related to cortical activity on areas of importance for action-planning, decision making and sustained attention. Taken together, they are in accordance with the Negativity Bias but do not support the Positivity Effect. The methodology used was able to identify age-related differences in cortical activity during emotional mnemonic processing and may be interesting to future investigations.

## Introduction

Memory – the ability to acquire, retain and utilize information and knowledge – is a fundamental process that allows learning and adaptive behavior, since the organism can use its previous experiences to select the most appropriate behavior for the upcoming situation. This ability has originated as an adaptation to a complex and constantly modifying environment [1], [2].

Working memory [3], [4] is the type of memory that keeps information as long as it is being used for comprehension, reasoning, planning and problem solving, for instance [5]. Working memory is also fundamental to maintain the attentional focus on one stimulus while its information is being manipulated. One of the subsystems of this type of memory is the visuo-spatial sketchpad, responsible for processing visual and spatial information and important in spatial orientation [6].

One of memory's main modulators is emotion, which can be defined as a psychophysiological response of value attribution to a stimulus [7] or as a subjective experience accompanied by organic and behavioural displays [8]. The interaction between these two features has been reported in several studies and includes the overlap of brain structures such as the hippocampal formation and the amygdala [9]–[11]. Emotional memory is, therefore, the one influenced by emotions, motivations and moods. Since information is more easily consolidated when it has an emotional connection to the individual, this interaction can be considered adaptive, for it allows the keeping of information that are relevant to its survival in a more efficient way [12], [13].

Emotions can be classified into one of three valences, “negative”, “positive” or “neutral” [14]. Negative emotions are better known by science because they produce the greatest physiological manifestations and are more mandatory for the survival of the organism [15], while positive and neutral emotions elicit lesser responses. Emotions generate an interpretation of the stimulus, which leads to differentiated recollection [14].

Of all behavioral displays of emotions, facial expressions are considered one of the most important and basic ways to externalize emotions [16], [17], especially to social species such as humans [18]. Facial expressions of emotions are interesting stimuli to cognitive tasks because they are biologically and evolutionarily important, quickly processed and some of them are universally identified [19], [20].

Studies on ageing have indicated a decline of several cognitive functions, including memory, which is among the most frequent complaints made by the elderlies. Some possible reasons are linked to the thinning of cortex layers, especially in the prefrontal lobe [21]; changes in the blood supply for the brain [22]; slowing of the processing speed; and reduction in the efficiency of attentional and inhibitory mechanisms [23]. Emotional memory characteristics change across lifespan, for instance, the influence of each emotional valence. On the one hand, the Negativity Bias hypothesis predicts that young people will focus their attention and better remember negative events [24], [25], on the other hand, the Positivity Effect hypothesis states that older adults will show the opposite pattern thus remembering positive events better, since they are aware that they will not live much longer [26]–[28]. Both hypotheses have been demonstrated through behavioral data with visual stimuli, with history facts and in both long-term and working memory.

The Positivity Effect Hypothesis is based on the Socioemotional Selectivity Theory [26] that states that the goals of each person are formulated according to his/her temporal context, which is the way their remaining life time is perceived. While young people perceive their future as a long period of time ahead of them, and are thus motivated by the pursuit of knowledge, the elderly are aware that there is not much time left and put their cognitive resources on the search for emotively meaningful experiences. In line with these studies, it is expected that not only behavior, but also cortical activity of young and older adults will present differences during emotional memory processing.

Behavioral measures are useful to elicit cognitive processes. The Spatial Delayed Recognition Span Task (SDRST) efficiently requires the use of visuo-spatial working memory [29]. When the stimuli used differ in their emotional valence, differences in performance may represent the interaction between emotion and memory [30]. These patterns are deeper investigated with the aid of electroencephalographic (EEG) measures, since similar behaviors may be generated by different cortical activations [31].

The Positivity Effect Hypothesis has not yet been tested in visuo-spatial working memory tasks. Just as well, even though it is known that the young and elderly brains show unequal activity [32]–[34], it has not yet been investigated how – and if – the Positivity Effect leads to changes in the cortical activity between young and older adults during emotional memory processing. Thus, the present study aimed at investigating (1) if the Positivity Effect happens in a visuo-spatial working memory task with facial expressions as stimuli, and (2) if it leads to differences in the cortical activity of young and older adults performing that task.

## Materials and Methods

### Participants

This study included twenty-seven young [(YA): 13 males; 18 to 25 years old] and twenty-five healthy older [(OA) adults: 14 males; over 60 years old] with more than 12 years of education. All were right-handed volunteers; had no history of neurological or psychiatric episodes; were not making concomitant use of psychotropic medication; and were naïve about the aims of the study. A written informed consent in accordance with the ethical guidelines for research with human subjects (196/96 CNS/MS Resolution) was obtained from all participants. The study was approved by the Human Subjects Ethics Committee of the Health Sciences Faculty of the University of Brasilia. All participants had normal or corrected-to-normal vision and hearing.

An evaluation of previous medical history and two neuropsychological screening tests to assess global cognitive function were applied for all subjects. We used Brazilian versions of the Mini-Mental State Examination (MMSE; [35], [36]) and of the Philadelphia Brief Assessment of Cognition (PBAC; [37]). Additionally, the Geriatric Depression Scale (GDS; [38]) was used to assess depression or the potential for depression in older adults. These neuropsychological tests were used as an additional exclusion factor as well as a way of confirming that any differences found between groups on the memory SDRST were not due to differences in other cognitive features.

### Spatial Delayed Recognition Span Task conditions

**Figure 1:**
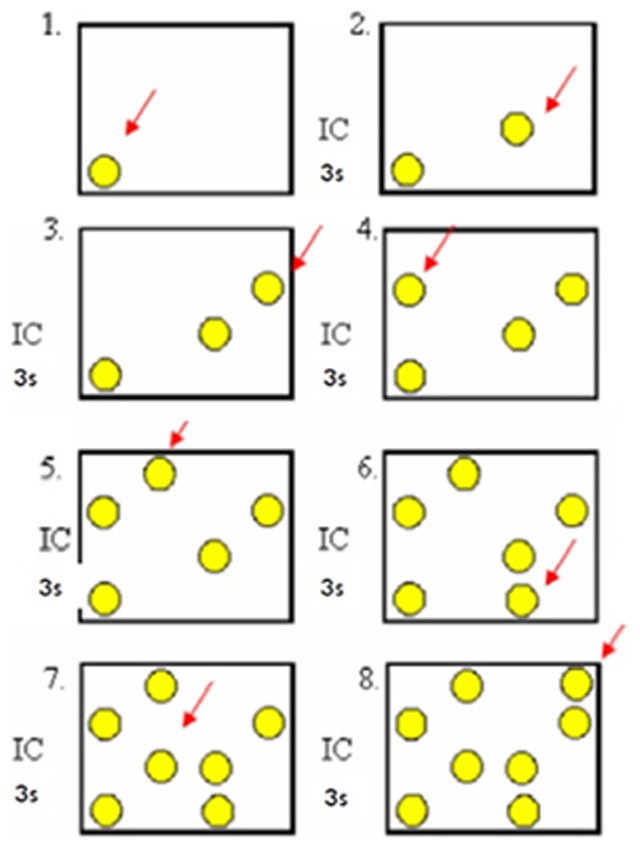
Experimental design for the Spatial Delayed Recognition Span Task. The full procedure included 40 trials of up to a maximum of 8 identical stimuli, 10 trials for each stimulus category (geometric pictures, positive, neutral and negative faces). The participant had to identify the stimulus in the new location. There was a 3-second interval of configuration (IC) between each new stimulus is presented on the touch screen.

A computer-based version (Delphi language, computational program SYSMEN) of this task was presented to the subjects on a touch screen monitor (LG Studio Works 440, Microtouch, 17′). Participants had to discriminate a novel location of a stimulus among an increasing array of identical stimuli presented sequentially in various locations within the same trial (for more specific details, see [29], [39]). The first stimulus was presented in one of the 16 possible positions randomly selected by the computational program and the participant had to press it. It would then disappear for 3 seconds and reappear in the same location and in a new one. The subject had to press the stimulus in the new location (Figure 1). The number of stimuli would increase up to a maximum of 8 or until a mistake was made. Correct answers led to an acute auditory feedback signal, and wrong answers, to a bass auditory signal.

Stimuli could be either geometrical pictures (colorful squares and circles of 2 cm diameter/high) or photographs (3 cm×4 cm) of facial expressions performed by adult models, manipulated to only show the face, with no interference from hair or other body parts. These photos could be neutral, positive (happiness expressions) or negative (anger expressions). Therefore, each stimulus belonged to one of four mutually exclusive categories: geometric, neutral, positive or negative. Participants performed a 10-trial block for each stimulus category.

### Data acquisition and processing

EEG data were obtained from 21 scalp channels placed in accordance with the 10–20 international system. These electrodes and the two reference electrodes (on the right and left mastoids) were fixed by a conductive paste (Ten20,Weaver and Company, USA) after the scalp sites had been previously prepared with an abrasive gel (Nuprep, Weaver and Company, USA). Continuum records were made with a NeuroSpectrum 4EP system (Neurosoft, Russia) at a sampling rate of 2000 Hz and impedances kept lower than 5 kΩ.

Data were processed using Matlab scripts under EEGLAB (v. 9.0.4.5; [40]; http://sccn.ucsd.edu/eeglab/) that digitally separated the recording into non-overlapping epochs time-locked to each stimulus category. These epochs were decomposed into independent components by an infomax algorithm (ICA; [41]). Topographic maps were generated after the independent components that represented eye movements, blinking and pulsation were removed from the original data.

### Procedure

Participants were received in the recording room, equipped with a Faraday cage (259 cm×223 cm×396 cm). They read and signed the written informed consent. After that, they answered to MMEE, PBAC (all volunteers) and GDS (only OA volunteers), in that order. Then, they were invited to sit comfortably in front of the touch screen, which was positioned within the reach of each volunteer. Electrodes were installed and lights and noises were reduced. Participants were asked to keep their eyes closed and to not move for a while. This part of the procedure lasted 60 seconds and intended to record the cortical activity baseline for each subject. The instructions for the test were read and participants answered to one training session with the aim of verifying if the test rules had been understood. Instructions were kept constant for all subjects.

The first 10-trial block of the task always presented geometric pictures, but the order of the three 10-trial blocks for facial categories was pseudo-randomized across participants. Each block was separated from the previous by a 30-second closed-eye rest. Computer software registered correct responses, wrong responses and response time for each answer given. The time of execution of the task varied according to each participant's response time, but all procedures (including neurocognitive tests) did not last more than two hours. After all four stimulus categories were performed, subjects answered questions about their reactions to the task and received the complete explanation about the objectives of the research.

### Statistical analysis

Performance was obtained through two measures: mean of scores, calculated as the mean of correct choices before a mistake in each 10-trial block; and mean of response time for all answers given in each 10-trial block. Those values were analyzed (SPSS v 18.00; SPSS, Inc., Chicago, IL, 2009) by a mixed design ANOVA with the factors Age Group (YA or OA; between-subjects) and Stimulus Category (geometric, neutral, positive and negative; within-subjects). *Post hoc* tests were conducted with dependent-samples t-tests. Significance was adjusted with Bonferroni method and defined as a *p* value of less than 0.05. EEG data analysis was made with the parametric statistical tools of the open source EEGLAB platform and significance level was adjusted with the False Discovery Rate method [42] and defined as a *p* value of less than 0.05.

## Results

### Behavioral results

Demographic and clinical data of the samples are given in Table 1. Educational level (years) did not differ between age groups. Overall, mean scores of neuropsychological tests (MMSE, PBAC and GDS) were within the expected range in the healthy Brazilian population [35], [37], [43]–[45]. However, OA had significantly lower MMSE and PIBAC scores than YA. GDS scores did not reveal depression or potential for depression.

**Table 1:** Demographic and clinical characteristics.

	YA (n = 27)	OA (n = 25)
Age, years	21.4±2.1	69.6±6,2
Education, years	14.19±1.71	14.38±4,17
MMES	61.14±6.10*	45.81±5.18
PBAC	55.79±2.91*	50.33±4.45
GDS	—	4.73±2.53

YA = Young adults; OA = Older adults; MMES = Mini Mental State Examination; PBAC = Philadelphia Brief Assessment of Cognition; GDS = Geriatric Depression Scale. Values are mean±SD. * p<0.001 vs. OA (Student's t test).

Behavioral results for SDRST revealed significant statistical differences in the mean of scores between age groups (YA>OA; F_1,49 = _42.787; p<0.001) and between stimulus categories (F_3,147 = _4.093; p = 0.008), but not for the interaction between the factors (F_3,147 = _0.225; p = 0.879). Pairwise comparisons showed that positive faces elicited lower scores than negative faces (p = 0.004) (Table 2).

**Table 2:** Scores and response time of young and older adults on the SDRST according to the stimulus category.

	Mean of scores		Mean of response time (ms)	
Category	YA^#^	OA	YA	OA
Geometric	7.359±0.665	5.917±1.022	1420.421±283.008•	1643.194±368.314•
Negative faces	7.558±0.438	6.195±1.065	1337.423±308.327	1482.827±356.490
Neutral faces	7.416±0.696	5.898±1.216	1294.654±307.063	1460.567±344.271
Positive faces	7.270±0.710*	5.845±1.021*	1398.438±324.897"	1507.833±353.360"

SDRST = Spatial Delayed Recognition Span Task; YA = Young Adults; OA = Older Adults; Values are mean±SD; ^#^YA>OA, p<0.001; *Positive<negative, p = 0.004; • Geometric>all other categories. "Positive>neutral, p<0.010.

Regarding the mean of response time, there were significant statistical differences between stimulus categories (F_3,144 = _13.610; p<0.001), but not between age groups (F_1,48 = _4.451; p = 0.069) or for the interaction between factors (F_3,144 = _1.842; p = 0.142). Pairwise comparisons showed that response time mean was greater to geometric pictures than to the facial photos (p<0.010) and to positive faces than to neutral faces (p = 0.008) (Table 2).

### EEG results

In general, cortical activity was predominantly registered in prefrontal and frontal cortices and in the central region of the scalp. Besides that, topographic maps indicated that activity was higher on the left hemisphere.

**Figure 2:**
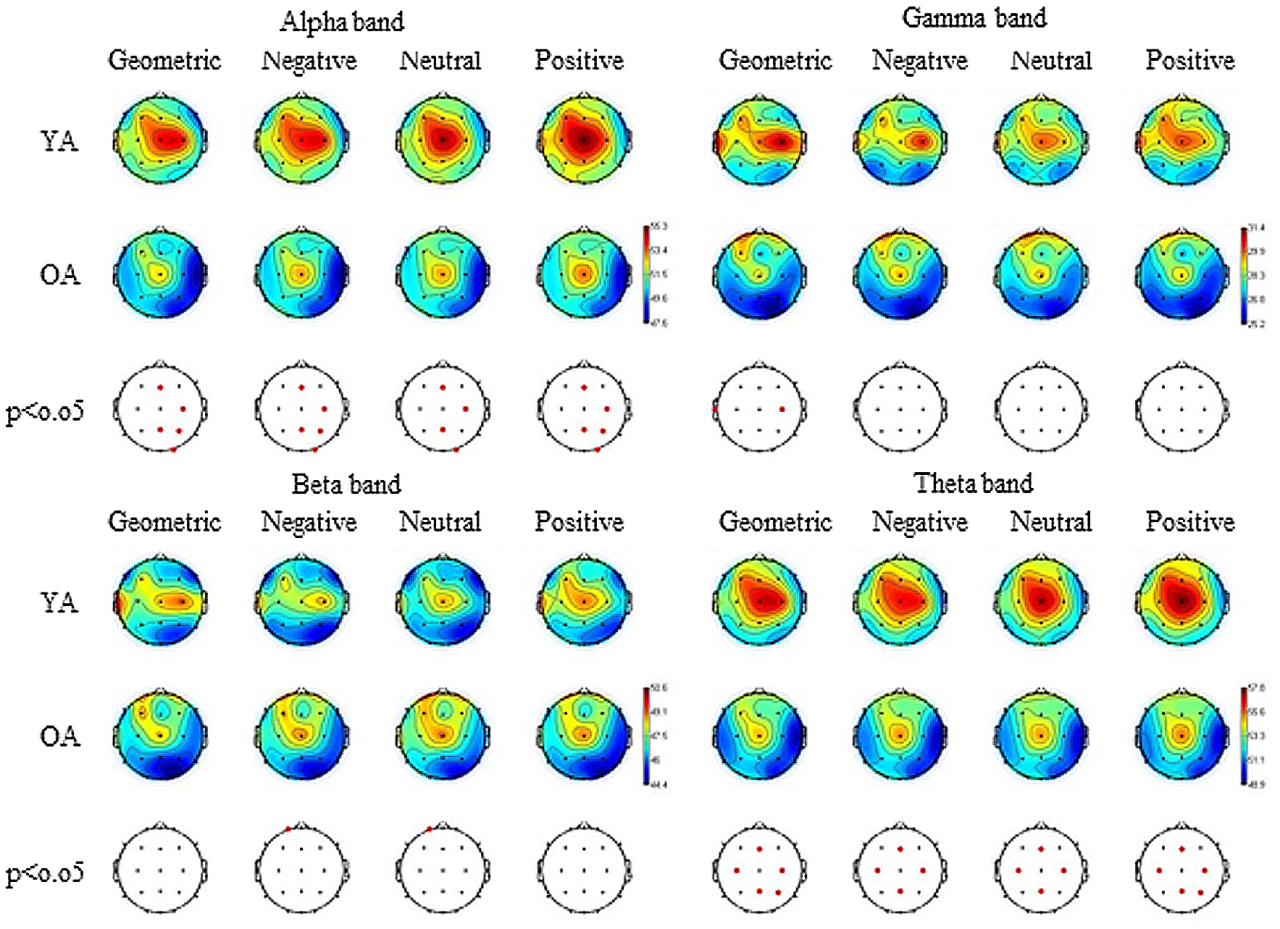
Relative topographic power spectrum distribution for specific bands with each stimulus category and age group. Stimulus categories are: geometric pictures, negative, neutral and positive faces. Age groups are: young adults (YA) and older adults (OA). Alpha activity: 8 to 13 Hz, Beta activity: 13 to 30 Hz, Gamma activity: 30 to 70 Hz, Theta activity: 4 to 8 Hz. Red dots indicate significant statistical differences (p<0.05; parametrical tests) related to age groups in electrode location.

**Figure 3:**
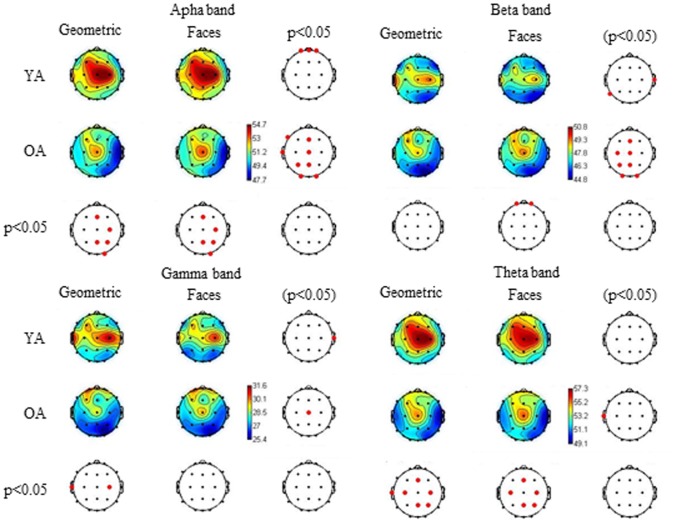
Relative topographic power spectrum distribution for specific bands with type of stimulus and age group. Types of stimulus are: geometric pictures and facial photos. Age groups are: young adults (YA) and older adults (OA). Alpha activity: 8 to 13 Hz, Beta activity: 13 to 30 Hz, Gamma activity: 30 to 70 Hz, Theta activity: 4 to 8 Hz. Red dots indicate significant statistical differences (p<0.05; parametrical tests) related to age groups and to types of stimulus in electrode location.

EEG data were filtered and divided into the traditional frequency bands: theta (4–8 Hz), alpha (8–13 Hz), beta (13–30 Hz) and gamma (30–70 Hz). *Theta*: YA had a higher activation on the central regions of the scalp, surrounding electrode *Cz*, in all four stimulus categories when compared to OA (Figure 2). Comparisons between the stimulus categories showed only one statistical difference in OA, with a higher activation for facial stimuli on the electrode *T3*. (Figure 3) *Alpha*: YA had a higher activation on the right hemisphere, in all stimulus categories, when compared to OA (Figure 2). For YA, facial pictures elicited higher activation in the prefrontal region, compared to geometric pictures. For OA, however, higher activation with facial stimuli was presented in the midline of the scalp, in the occipital region and in the left temporal regions (Figure 3). *Beta*: Few differences were obtained between age groups, with higher activation for OA on the left prefrontal region in negative and neutral categories (Figure 2). For YA, there was a higher activation for geometric pictures in specific regions of electrodes *T4* and *T5*. For OA, however, larger differences were obtained, with higher activation for facial pictures in the occipital region and in the midline region of scalp, especially in the left hemisphere (Figure 3). *Gamma*: YA and OA differed only on electrodes *T3* and *C4*, with higher activation to YA (Figure 3). For YA, only electrode *T4* showed difference between geometric pictures and faces, with higher activation for the first category. As for OA, the only difference was on the electrode *Cz*, with higher activation for facial stimuli (Figure 3). No statistical differences were found in any band when comparing negative and positive facial stimuli for YA or for OA.

## Discussion

This study explored EEG data of young and older adults performing a visuo-spatial working memory task with processing of emotional facial expressions. The goals were to investigate whether the Positivity Effect was present in this type of memory and if it would lead to differences in the cortical activity of the age groups during the task.

### Behavioral data

Analysis of performance on SDRST showed that OA had fewer correct responses compared to YA in all stimulus categories (geometric pictures, neutral faces, positive faces and negative faces). These results are in accordance with a number of studies suggesting that healthy ageing leads to deficits on different cognitive domains, including working memory [26], [46]. Nevertheless, participants of both age groups showed the same performance pattern when the stimulus categories were compared: faces improved performance compared to geometric pictures and negative faces improved performance compared to positive faces.

One possible explanation for the better performance in the facial categories is that faces consist of a more biologically and socially important stimuli, so their cognitive processing receives more resources [19]. Additionally, studies have shown that facial detection is quicker than detection of other types of stimuli [47], [48], mainly because of the Fusiform Face Area [49]. Another hypothesis is the great familiarity that people have with this kind of stimulus, which enhances the efficiency of its processing [50], [51].

Considering the emotional valence effects, better performance with negative faces compared to positive faces supports the Negativity Bias Hypothesis, which states that due to their larger influence on the adaptive value of an individual, negative events will be more efficiently remembered. Besides that, among facial expressions, negative ones receive more attention, are detected faster and generate a prompter behavior response, since they indicate places to be avoided, imminent aggressive behaviors and possibility of contamination [19], [52]. Negative facial expressions attract more attention to their location than other valences [53]–[56], which can also have facilitated performance in the negative category.

Additionally, it has been shown that the emotional state which an individual is in influences his cognition, with negative emotions tending to enhance it [57], [58]. In this direction, several participants of the present study reported, at the end of the task, positive feelings, such as happiness, while viewing the positive faces and negative feelings, like anger, while viewing the negative faces and this may also have contributed to the better performance in the negative category.

Nevertheless, the memory enhancement in both young and older adults for negative stimuli found in the present study does not support the Positivity Effect Hypothesis. This is in line with other research [59], in which numbers and words of different emotional valences were presented to young and older adults and it was asked for them to indicate if the numbers were even or odd. After the task, participants were asked to recall the words presented. Although the elderly remembered more positive words than young adults, their scores during the task were the same, which indicates that both age groups were equally affected or distracted by the emotional words. This may have happened in the SDRST used in the present study, since information to be remembered was the location of the stimulus and not the image itself. It is worth pointing out that other studies have also found no evidence that older adults are better in remembering positive stimuli (e.g. [60], [61]).

### EEG data

Electrophysiological findings showed greater activation in prefrontal and frontal cortices as well as the central and parietal regions of the scalp, which are areas related to working memory [5], [62]. Prefrontal cortex is also related to improving maintenance of a facial stimulus in working memory despite of distracting factors [63], to attentional mechanisms [64], to utilization of previously learned rules to perform a task [65], and to affective processing (see [66]). In a previous research [67], the authors applied a spatial working memory test in rodents and measured cortical activation in the prefrontal cortex and in the hippocampal formation. They found that there was synchronization between these regions in alpha and in theta frequencies, indicating that these bands are important to integrating brain structures. This means that they are necessary to the accordance between registering spatial information and making a decision.

Participants showed higher theta band activation on the midline region of the scalp to all four stimulus categories. This pattern was seen in a previous study [68] and is related to attention, concentration mechanisms and mental effort [69], which are abilities involved in the successful solving of SDRST. Besides that, theta band is fundamental to integrating regions during mnemonic processing [70] (and see [71]). Also, theta band on the frontal area is involved in the maintenance of information in working memory and it increases with the cognitive load of the task [64]. Patterns of activation found for gamma and theta bands were similar. During mnemonic processing, gamma and theta bands work together [72] and coherence of phases has been show to predict performance [73]. Superposition of gamma and theta frequencies also seems to be important to the definition of the maximum number of items to be kept in working memory [74]. Besides that, gamma band in the prefrontal cortex and in the left temporal region is related to codification of visual stimulus [71], to the maintenance of spatial information in working memory and to attention [75], [76].

Topographic maps generated in this study indicate that cortical activity was somehow higher in the left hemisphere. Usually, visuo-spatial tasks lead to right-hemispheric asymmetry, while verbal processing tasks lead to left-hemispheric asymmetry [58], [68], [70], [77]–[79]. Thus, although in SDRST, participants were required to remember the spatial location of the stimuli, they may have made use of intern mechanisms of verbalization in order to complete the task [80], [81]. This strategy produces associations between stimulus characteristics, which make its maintenance in working memory more efficient [82]. Higher theta activation on the left parietal region had already been shown [75] in a task where participants had to indicate whether the item had or had not been presented beforehand. It was suggested that theta frequency in left parietal region was related to working memory, to action planning and to decision making. SDRST used in the present study requires all those abilities, so it makes sense that those results [75] were also found here.

Differences were found between activation for facial and for geometric stimuli. Older adults showed a higher activation for faces in the left frontotemporal region. This is in line with a previous study [82] in which the authors obtained greater activation for working memory tasks with facial stimuli on the left frontal region and a higher activation for the same task with non-facial stimuli on the right one. In another research [76], alpha band activation in tasks where subjects had to remember a face identity was related to focusing attention in a certain part of the task and inhibiting not important parts. Young adults, in the present study, exhibited a higher alpha activation on the pre-frontal region, which is in accordance with previous results [83] that compared cortical activation during working memory tasks using facial stimuli and words and showed that the first kind elicited higher activation in pre-frontal and parietal regions. This electrophysiological pattern may have contributed to the greater response time elicited by geometric stimuli in the present study, since alpha activation in this region is important to the efficient utilization of a previously learnt rule to solving a task [65].

The present study found no differences between cortical activation for positive and negative faces, which suggests that they are processed in similar brain regions. There are two theories to explain laterality patterns in emotional processing. The Right Hemisphere Hypothesis states that all emotional valences are processed predominantly by this hemisphere. The Emotional Valence Hypothesis, in contrast, says that positive emotions are processed in the left hemisphere and negative in the right hemisphere [80], [84], [85]. It is important to highlight that these hypothesis are more often tested in tasks where information to be kept is the image itself and not its location, like is the case of SDRST. It is possible that different cognitive mechanisms generate different activation patterns [86].

Results for alpha and theta bands showed that there was a greater activation on the frontal and central regions of the scalp for young adults in the four stimulus categories when compared to older adults. This pattern may be related to the lower performance of older adults, since these bands on these cortical sites are important to the efficiency of sustained attention and of inhibitory mechanisms [87], [88]. These are fundamental abilities to different cognitive domains and to the successful performance on SDRST, meaning that older adults may not have been sufficiently able to focus on the task, to prepare themselves for the upcoming trial and to efficiently decide the correct choice.

The Compensation-Related Utilization of Neural Circuits hypothesis (“CRUNCH”; [89]) predicts that even though both young and older adults will recruit more cognitive resources as task load increases, senior brains will show overactivation at lower and medium levels of task demands, as a way to compensate for age-related losses on processing efficiency, poor strategies or atrophy, while achieving behavioral output similar to the younger brain [90]. When facing a more difficult task, older adults will reach a ceiling of resources, while young adults will keep increasing cortical activity, which will lead to underactivation of the elderly brain when compared to its young counterpart and also to a lower performance.

Other studies have investigated patterns of activation of young and old adults with memory tasks, such as indicating whether a new letter was present on the previous set of letters, if the position of a new circle was the same as the previous presented [91], or performing the *n-back* task [92]. The results of the present study showed that, in general, young adults had better performance and higher activation than older adults in every stimulus category, which indicates that the task was highly difficulty. This is in keeping with other studies that suggest that SDRST is more demanding than other memory tasks since it presents an increasing number of items to be remembered and requires relational representation and flexible memory expression [93]–[96].

The only different case was seen on the beta frequency band, since older adults showed a higher activation than young adults on the left prefrontal region in negative and neutral stimulus categories, which is in line with previous studies demonstrating that this site shows the most pronounced evidences for overactivation and compensation [89]. This band is related to the maintenance of information in working memory, being important to the coordination of different cortical regions and to the internal representation of the stimulus in each new occurrence [69], [71]. Topographic maps indicate that the left asymmetry seen for young adults in this frequency band is less evident in the prefrontal region of the elderlies. This seems to be in line with the Hemispheric Asymmetry Reduction in Older Adults model (“HAROLD”; [32]) that states that older adults will show a more bilateral activation in the prefrontal regions when comparing to their young counterparts. This would happen as a way of counter-acting age-related cognitive deficits. In a recent study [90], it was suggested that the HAROLD model is a special case of the CRUNCH hypothesis since it is an age-related compensatory process that happens in specific regions.

In conclusion, regarding the objectives of this research, the behavioral results do not support the Positivity Effect in a visuo-spatial working memory task with emotional facial expressions, although differences in the cortical activity between young and older adults were found. Taken together, the results of this work contribute to the characterization of the relationship between cognitive processes and each emotional valence across lifespan. Importantly, the methodology chosen was able to identify age-related differences in cortical activity during emotional mnemonic processing and may be used in future investigations.

Future studies should also evaluate how individual differences in aspects such cognitive ageing, anxiety levels [97] and laterality patterns [98] influence memory and emotion interactions in the elderlies. It may also be accessed cortical activity, in both young and older adults, when different facial expressions of the same valence are used as stimuli, since previous studies have showed that they may [50] or may not [52] lead to behavioral differences. To extend the discussions about the CRUNCH hypothesis, EEG analysis in this working memory task could be compared when the cognitive load is low (1 to 4 stimuli on the screen) and high (5 to 8).
